# Two Cases of Oculofaciocardiodental (OFCD) Syndrome due to X-Linked BCOR Mutations Presenting with Infantile Hemangiomas: Phenotypic Overlap with PHACE Syndrome

**DOI:** 10.1155/2019/9382640

**Published:** 2019-12-28

**Authors:** T. M. Morgan, J. M. Colazo, L. Duncan, R. Hamid, K. M. Joos

**Affiliations:** ^1^Vanderbilt University School of Medicine, Department of Pediatrics, Nashville, TN, USA; ^2^Vanderbilt University School of Medicine, Medical Student, Nashville, TN, USA; ^3^Vanderbilt Eye Institute, Vanderbilt University Medical Center, Nashville, TN 37232, USA

## Abstract

**Background:**

Oculofaciocardiodental (OFCD) syndrome is due to mutations in BCOR (BCL-6 corepressor). OFCD has phenotypic overlaps with PHACE syndrome (Posterior fossa anomalies, Hemangioma, Arterial anomalies, Cardiac defects, Eye anomalies). Infantile hemangiomas are a key diagnostic criterion for PHACE, but not for OFCD. A previous study reported two cases of infantile hemangiomas in OFCD, but the authors could not exclude chance association.

**Case Presentation:**

We describe two novel cases of female patients (one initially diagnosed with PHACE syndrome), both of whom had infantile hemangiomas. Ophthalmological findings were consistent with oculofaciocardiodental (OFCD) syndrome. Upon genetic testing, these two females were determined to have X-linked BCOR mutations confirming OFCD syndrome diagnoses.

**Conclusion:**

These case reports add support to the hypothesis that infantile hemangiomas may be a feature of OFCD. BCOR may potentially be within a pathway of genes involved in PHACE syndrome and/or in infantile hemangioma formation.

## 1. Introduction

Oculofaciocardiodental (OFCD) syndrome and Lenz microphthalmia due to mutation in BCOR (BCL-6 corepressor) are allelic, X-linked, multiple congenital anomaly disorders that exhibit sex-dependent, variable expressivity [1]. Specific hypomorphic mutations (typically c.254C>T; p.Pro85Leu) present as Lenz microphthalmia syndrome, a classic X-linked recessive neurodevelopmental and multiple congenital anomaly disorder affecting males only, with female carriers having no known phenotypic manifestation [2]. Null mutations in OFCD segregate as an X-linked dominant disorder with presumed male lethality, presenting postnatally only in females. Phenotypic expression of OFCD varies, to a large extent related to skewing of X-inactivation. The most consistent phenotypic feature in OFCD is congenital or early onset cataracts. The classic diagnostic tetrad present in about two-fifths of cases requires additional features including facial anomalies (cleft palate; septate nasal cartilage; long, narrow facies with arched eyebrows), cardiac defects (septal and others), and dental anomalies (radiculomegaly, oligodontia, and delayed eruption) [3].

A recently reported case series noted the presence of hemangiomas in two affected individuals [4]. One was a female with OFCD due to monoallelic BCOR frameshift mutation. The other was a male with a novel hemizygous BCOR mutation (c.4807A > C; p.Ser1603Arg) detected by exome sequencing. This was transmitted from his unaffected mother, shared by his affected maternal half-brother who had multiple capillary malformations but no hemangioma, and absent in his mother's two other sons. The affected half-siblings did not have classic Lenz microphthalmia, and their shared ocular phenotype was bilateral posterior embryotoxon without cataracts. In addition to hemangiomas in two of the 16 affected cases, the authors noted that a carrier female related to one of the cases also had a hemangioma. The authors reviewed previously published literature, describing a grand total of 95 cases with pathogenic BCOR mutation derived from 66 families. No other hemangioma was reported, though the literature review was restricted to cases with documented BCOR mutation.

Here, we report two additional females with OFCD and history of infantile hemangioma. In one of the cases (case 2), PHACE syndrome (Posterior fossa , Hemangioma, Arterial anomalies, Cardiac defects, Eye anomalies) was provisionally diagnosed, and subsequent marked ocular similarity to case 1 was diagnostically confirmed by BCOR sequencing [5]. Given the phenotypic overlap between PHACE syndrome and cases of BCOR syndromes presenting with hemangiomas, we sought to systematically compare and contrast the features of the two diagnoses.

## 2. Materials and Methods

We report here the clinical details of two cases of OFCD presenting with infantile hemangiomas. We then show how these, along with the aforementioned two previously reported cases of BCOR-related infantile hemangiomas (IH), may fit into the consensus clinical classification of PHACE syndrome. Such criteria are summarized as follows: “Individuals with IH of the face or scalp >5 cm in diameter and 1 major diagnostic criterion are considered to have definite PHACE. In addition, patients with large segmental IH of the neck, upper trunk, or trunk and proximal upper extremity who also have 2 other major criteria should be considered to have definite PHACE.” We presented a cross-tabulation of cases and PHACE criteria (Table 1). To further analyze, any molecular genetic studies of PHACE that might shed light on BCOR's candidacy for this diagnosis, we performed a gene set enrichment pathway analysis (GSEA). This study was conducted in accordance with the principles of the Declaration of Helsinki, and the study, as a de-identified case report of three or fewer individuals, was exempt by formal policy from review by the Vanderbilt University Institutional Review Board (policy I.B.1, last revised on April 28, 2011, https://www.vumc.org/irb/).

## 3. Results

### 3.1. Case 1

**Figure 1:**
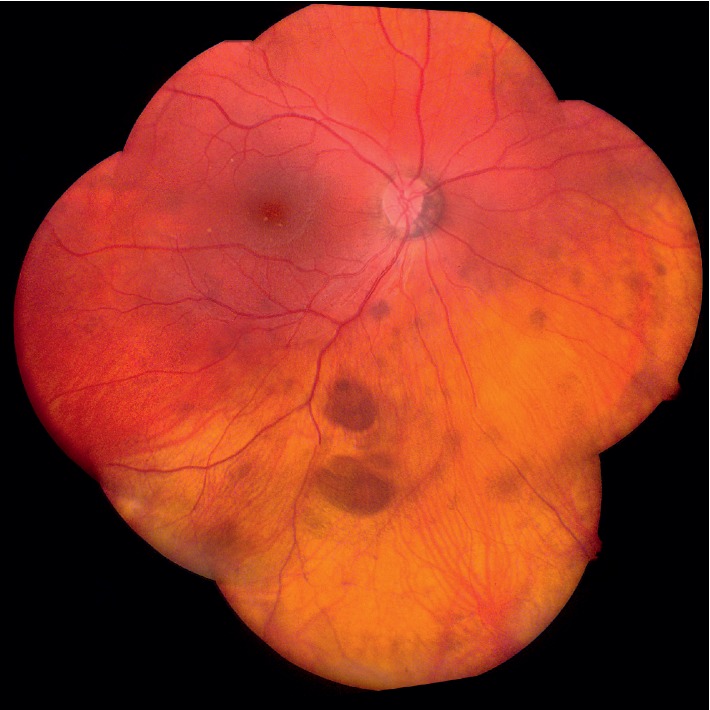
Ocular phenotype (case 1). Multiple lesions of congenital hypertrophy of the retinal pigment epithelium (CHRPE) shown inferiorly and pigmentation near the optic nerve in the right eye of a female with aphakia after surgical removal of congenital cataracts.

**Figure 2:**
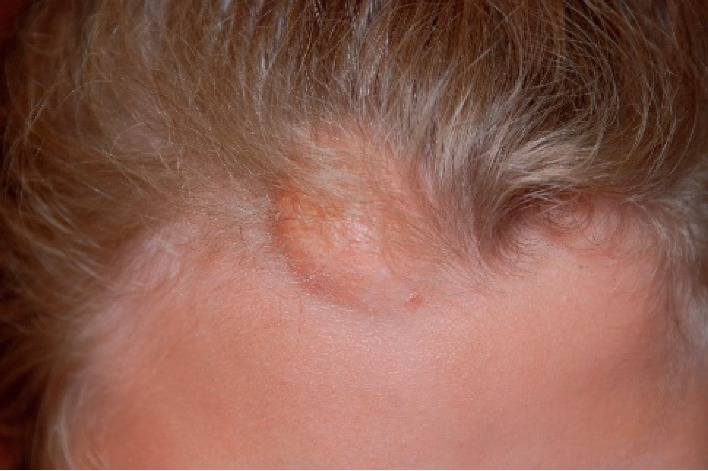
Involuted frontal scalp hemangioma (case 1).

This is a 15-year-old female who first presented for medical care to an outside pediatric ophthalmologist with bilateral congenital cataracts that were surgically removed at one month of age, with removal of reproliferation secondary membranous cataract from the right eye at 4 months. She also had a heart murmur which resolved and a “birthmark on her forehead.” At age 3.5 years, she was started on topical 0.5% timolol and then referred to the Vanderbilt University Medical Center (VUMC) glaucoma service for consultation and management of increased intraocular pressure (IOP) bilaterally (37 mmHg OD and 36 mmHg OS) and photophobia in the setting of aphakia. Bilateral early secondary glaucoma/ocular hypertension, bilateral inferior multifocal congenital hypertrophy of the retinal pigment epithelium (CHRPE) with pigmentation adjacent to the optic disc in the right eye (Figure 1), and a scalp hemangioma (Figure 2) were noted. Neuroimaging was not performed. She was initially managed with topical glaucoma medications which controlled the intraocular pressure in the right eye. However, over the next couple of months, her glaucoma remained uncontrolled in the left eye despite advancement to maximal medical therapy, so she underwent placement of an anterior chamber Baerveldt shunt and complete pars plana vitrectomy to prevent shunt blockage. She developed inflammation and vitreous bands requiring steroids, repeat pars plana vitrectomies with membranectomy, and revision of the shunt. Her left eye became quiet and her IOP has remained controlled to date in both eyes since age 4.5 years with supplemental topical glaucoma medications for both eyes.

After a few years, when she was 8 years old, due to her bilateral multifocal CHRPE diagnosis, she was referred to genetics by her glaucoma specialist to be evaluated for possible Gardner syndrome, with no family history of this disorder [6]. *APC* mutation testing was negative. At that time, she was in third grade and doing well.

**Figure 3:**
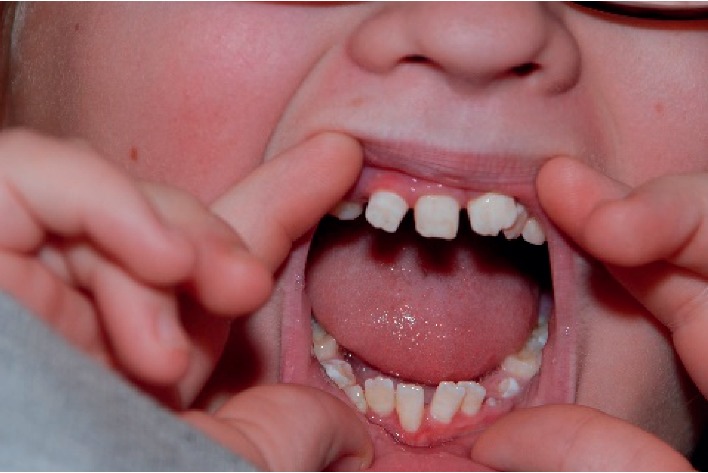
Dental phenotype (case 1).

A few months later, she was seen again in the genetics clinic due to her mother's concern for a possible genetic syndrome. At that time, the patient's re-evaluated developmental history was significant for a history of VSD, dental anomalies (persistent baby teeth, fused baby teeth, delayed eruption of secondary teeth, and long roots) (Figure 3), flat feet, a high-arched palate, and facial features that do not resemble other family members. Birth history was notable for being a 3.43 kg, 52.0 cm product of full-term uncomplicated pregnancy to a 20-year-old gravida 2, para 2 female. Additional past medical history included migraine headaches and recurrent otitis media, with pressure equalization (PE) tube placement at 4 years of age as well as tonsillectomy and adenoidectomy.

Family history was documented by a 4-generation pedigree. Her sister, 5 years younger, had strabismus, and was otherwise normal in health and development. Mother had a history of unilateral amblyopia, patched as a child. Father had hypertension and history of a heart murmur as a child. Maternal aunt with two sons and a daughter were all alive and healthy. Father's mother had a colon polyp at age 28, but no history of APC or other genetic testing or counseling, and no further details are known. Father had two maternal half-siblings, one a female with history of a murmur and the other a male with history of extra teeth. Father's father was alive and well. Both of her parents were of mixed European ancestry.

A complete physical examination was performed. On general appearance, patient was dysmorphic and wearing aphakic glasses. Pertinent positives included a bump on her forehead that could represent an involuted hemangioma, high-arched palate, irregular tooth development and location, and positive clinodactyly but no polydactyly.

Based on the history and clinical presentation, OFCD was suspected. BCOR sequencing was performed, showing a c.776C>A;p.S259X monoallelic pathogenic mutation in exon 4, previously reported in OFCD. Clinically unaffected parents chose not to be tested after test result disclosure by a genetic counselor.

### 3.2. Case 2

**Figure 4:**
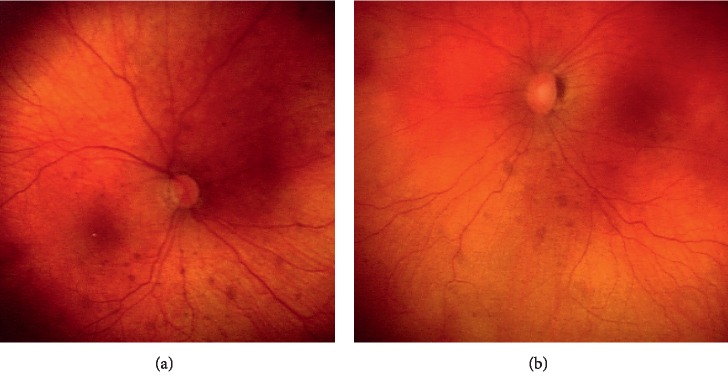
Ocular phenotype (case 2). Multiple lesions of congenital hypertrophy of the retinal pigment epithelium (CHRPE) present bilaterally ((a) right eye; (b) left eye) in a female with aphakia after surgical removal of congenital cataracts. Asymmetric increased cup to disc ratio was present in the left eye.

This is a 10-year-old female who first presented at age 4 days to Vanderbilt University Medical Center pediatric ophthalmology for bilateral congenital cataracts. She underwent cataract extraction and anterior vitrectomy at age 5 and 6 weeks. She required removal of reproliferation secondary membranous cataract in both eyes 2 months later. An additional two months later, she was referred to the glaucoma service for elevated IOP in the right eye (30 mm Hg). Examination under anesthesia revealed a hemangioma near her right eyebrow and a soft protuberance at her posterior scalp, IOPs in both eyes controlled on topical glaucoma medications required only in the right eye, bilateral multiple CHRPE (Figure 4), and axial eye lengths indicating left eye microphthalmia. Her early secondary glaucoma/ocular hypertension subsequently required the addition of glaucoma medications to the left eye. Both eyes were then controlled with glaucoma medications until age 17 months at which time she underwent an anterior chamber Baerveldt shunt and complete pars plana vitrectomy in the left eye. The eye required additional steroids, vitrectomy, and membranectomy 5 and 21 months later. Subsequently, the IOP has remained controlled bilaterally on topical glaucoma medications.

She also had an atrial septal defect confirmed by ECHO at 1 month of age, and concern for possible Wolff-Parkinson-White (WPW) syndrome by the ECG per her local cardiologist's note.

**Figure 5:**
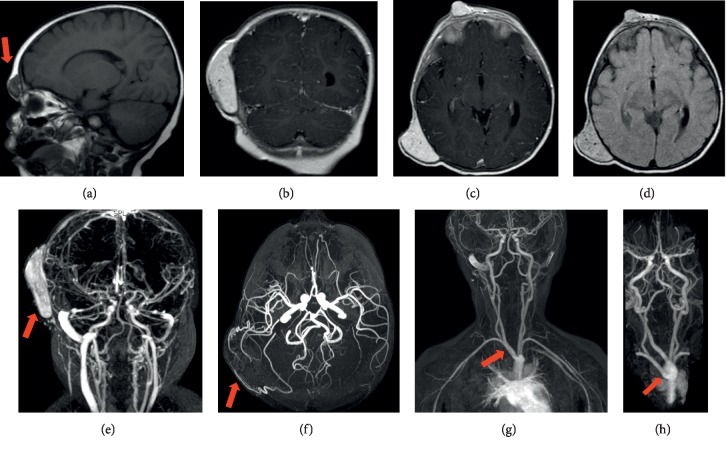
MRI/MRA images (case 2). (a) Sagittal MRI (T1) image of the brain showing a 1.2 cm diameter extracranial hemangioma on the right frontal scalp (red arrow). (b) Coronal MRI (postcontrast T1W) image showing the larger hemangioma on the right parietal scalp. (c) Axial MRI image (postcontrast T1W) showing both the smaller hemangioma on the right frontal scalp and the larger hemangioma on the right parietal scalp measured 2.9 × 2.3 cm axially. (d) Axial MRI image (SWI) showing both the smaller hemangioma on the right frontal scalp and the larger hemangioma on the right parietal scalp. (e) Head and neck MRA showing intracranial circulation and the large right parietal scalp hemangioma (red arrow). (f) Head MRA showing intracranial circulation and direct circulation (red arrow) to the right parietal scalp hemangioma. (g) Upper body MRA showing branching of upper body large arteries. (h) Zoomed in and angled upper body MRA showing the aberrant right subclavian artery (red arrow) coming directly off the aortic arch and not from the brachiocephalic artery (as it normally should). There is no observed right brachiocephalic artery.

When she was 11 months of age, pediatric neurology evaluated the vascular lesion on the parietal scalp and estimated its size to be 5 × 6 cm and specifically hypothesized PHACE syndrome. Due to this finding, she underwent brain MRI/MRA at 12 months of age which showed a large enhancing lesion of the right parietal scalp measuring up to 3.9 × 2.3 cm axially, interpreted as a likely hemangioma (Figure 5). There was an adjacent prominent intracranial draining vein in the right parietal region. A second smaller lesion was also present in the right frontal scalp with similar imaging characteristics. No intracranial abnormality was seen in association with the smaller lesion.

At 25 months, repeat imaging was performed, showing that the right parietal scalp mass contained multiple enhancing vessels with at least one feeding artery from the right external carotid artery. The right parietal lesion demonstrated diminished overall thickness measuring approximately 1.4 cm on the current exam compared to 2.2 cm in thickness on the prior exam. No communication with the intracranial vasculature was identified. The smaller lesion within the right frontal scalp was unchanged in size measuring approximately 1.2 cm in diameter. This lesion also demonstrated no definite communication with the intracranial vasculature. Imaging at this time also showed an aberrant right subclavian artery. The right external carotid artery supplied at least one arterial branch to the right parietal scalp mass, and all intracranial arteries were patent and without abnormality. Overall, these results showed that the right frontal and right parietal scalp lesions demonstrated no communication with the intracranial arterial system and a decreased size of the right parietal scalp lesion from previous imaging which may have represented early involution. She had appropriate growth, so endocrinopathies that may occur in PHACE were not suspected. She had no reported dental or hearing problems nor structural brain malformations.

She presented at age 9 years to the genetics clinic for confirmation of the probable diagnosis of PHACE syndrome. Due to these findings, a “definite diagnosis” of PHACE syndrome was given based on two forehead/scalp hemangiomas that grew rapidly as an infant and then resolved as well as history of an arterial anomaly (aberrant subclavian artery), cardiac defect, and eye anomalies (i.e., 4 of 5 major criteria for PHACE based on 2016 Journal of Pediatrics report by Garzon et al. on consensus diagnostic criteria [7]). During this appointment, it was noted that she had not had dental problems. The family history was notable for her mother having severe to profound congenital hearing loss diagnosed at six months of age and currently requiring hearing aids. Her father had a heart murmur in childhood. Her sister had childhood rheumatoid arthritis in the knee but was in remission.

**Figure 6:**
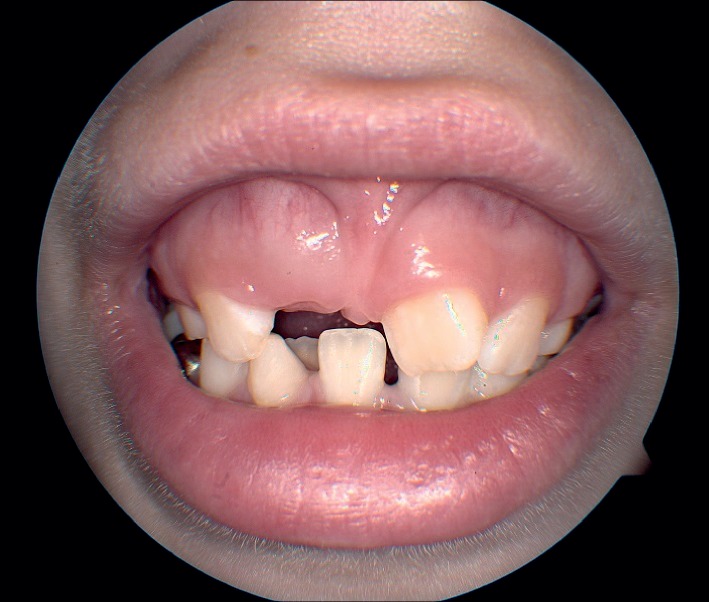
Dental phenotype (case 2).

During regular follow-up, her glaucoma ophthalmologist (KMJ) noted that the ocular phenotype, including congenital cataracts, glaucoma, and bilateral CHRPE raised strong suspicion for oculofaciocardiodental syndrome (OFCD) based upon similarity to the phenotype of case 1. Her parents also reported that she had long roots of her teeth with one missing tooth and first primary tooth loss at 6-7 years of age (Figure 6). Due to this important clinical observation and her cardiac history, genetic testing for this condition was performed, revealing a pathogenic monoallelic variant, c.2514del(G), p.Lys839Serfs∗17 consistent with X-linked OFCD. Her mother, father, and sister tested negative for the variant in DNA derived from peripheral blood lymphocytes.

**Figure 7:**
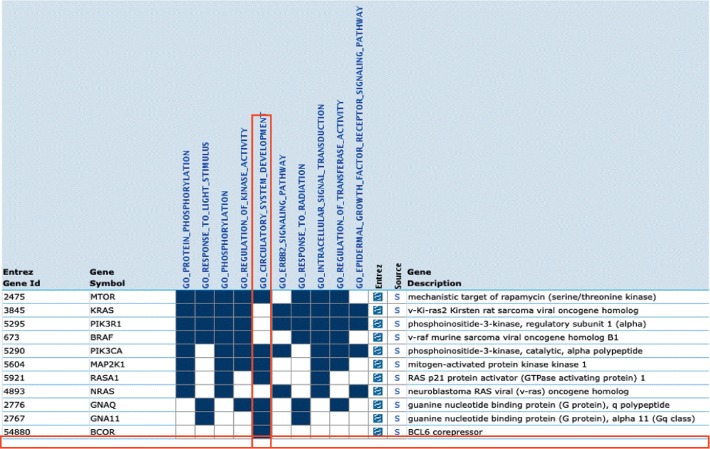
Gene set enrichment analysis. BCOR (red label, horizontal) was found to overlap with other genes in the pathway for “circulatory system development” (red label, vertical).

To further analyze any molecular genetic studies of PHACE that might shed light on BCOR's candidacy for this diagnosis, we performed a gene set enrichment pathway analysis (GSEA) (GSEA—Broad Institute [8, 9], http://software.broadinstitute.org/gsea/index.jsp) by adding BCOR to Sigel's (2018) gene list [10]. When BCOR is added among *BRAF*, *GNA11*, *GNAQ*, *KRAS*, *MAP2K1*, *MTOR*, *NRAS*, *PIK3CA*, *PIK3R1*, and *RASA1*, GSEA shows that BCOR overlaps significantly with other genes in the pathway of domain of “circulatory system development (Figure 7).”

## 4. Discussion

Infantile hemangiomas have not been labeled as a key characteristic of OFCD or other BCOR-related disorders. Along with two previously published cases, we report two new cases of infantile hemangioma in OFCD [4]. The precise characteristics of infantile hemangiomas including location and size are important to note, but such features may be poorly recalled as a “birth mark that went away” if the child is evaluated later in life. It may be informative to request and review photographs documenting the evolution of the vascular lesion, noting that early rapid growth is the key characteristic that differentiates an infantile hemangioma from other vascular birthmarks [11].

Due to this early involution and medical professionals and parents labelling the feature as a “birth mark,” infantile hemangiomas may be underreported in BCOR-related disorders. Although the precise incidence of infantile hemangiomas remains unknown, a reasonable estimate is that they occur in 4–5% of infants [12, 13]. Thus, even with the addition of the two cases reported here to the two found among 95 cases of OFCD drawn from 66 families, the prevalence of OFCD would not exceed statistical expectations (4/97 = 4.12%) [4]. However, infants do not usually get the type of infantile hemangiomas described in these cases, i.e., large (>5 cm) forehead/scalp hemangiomas. Furthermore, the two cases that we report were subject to selection and recall bias (though we knew of no other local cases to report). The epidemiology of OFCD is confounded by the same problems that attend infantile hemangiomas, because these develop and fade during infancy and often are dismissed when small as being harmless and temporary. Furthermore, these patients may be diagnosed with another disorder that has been more classically associated with infantile hemangiomas, such as PHACE.

PHACE syndrome's key feature is an infantile hemangioma, whereas the presence of other cardinal malformation is highly variable. Though there are well-accepted consensus diagnostic criteria based on pattern recognition, PHACE is ultimately a diagnosis of exclusion [7]. Aberrant subclavian artery is not rare in the general population (1.2%), but it is 20 times more frequent in PHACE. According to the census-derived diagnosis and care recommendation document, an “aberrant origin of a subclavian artery was the most common cardiovascular anomaly (present in 31/150 (21%) of subjects).” This is a major criterion for PHACE diagnosis. Combined with a large facial hemangioma, it establishes a “definite diagnosis” of PHACE. Congenital glaucoma has been reported in PHACE syndrome, so despite its apparent rarity in PHACE, its presence does not serve to exclude the diagnosis, and it may also present in Sturge-Weber syndrome, a diagnosis often confused with PHACE [14]. Microphthalmia does not assist in differentiating BCOR-microphthalmia syndromes from PHACE, as it is a minor diagnostic criterion for PHACE. Posterior fossa anomalies, when present, add weight to a clinical diagnosis of PHACE, but these can also be present in BCOR-microphthalmia syndromes, although rare. In addition, ocular coloboma can also be a feature of either PHACE or BCOR [15]. Overall, recalling that PHACE is a diagnosis of exclusion, exome or genome sequencing should be considered if no obvious alternative diagnosis is evident.

Dental anomalies may be somewhat helpful in deciphering OFCD from PHACE, as the only well documented tooth anomaly associated with PHACE is enamel hypoplasia, present in 28% of cases, but there has been no systematic survey and OFCD dental anomalies are not so distinctive that they provide high specificity for that diagnosis [7, 16].

In case 1, the diagnosis of PHACE was not suspected. However, it arguably could have been considered in the differential diagnosis, given the presence of a large forehead hemangioma, even if it did not fully occupy the frontonasal segment. The ocular findings included congenital cataracts, a minor diagnostic criterion for PHACE, as well as congenital hypertrophy of the retinal pigment epithelium (CHRPE), a posterior segment anomaly that could be counted as a major diagnostic criterion [10]. She also had a cardiac ventricular septal defect, another minor criterion for PHACE diagnosis. Thus, with a facial hemangioma, one major and two minor criteria, she would qualify for definite PHACE diagnosis, with the caveat that it is a diagnosis of exclusion [7]. Thus, both cases presented here could rightly have been clinically classified as having PHACE, and it is important to explicitly consider OFCD in the differential diagnosis of PHACE. Because patients with PHACE may have life-threatening but potentially treatable cardiovascular anomalies such as aortic coarctation or central nervous system complications such as moyamoya vasculopathy [17], it is important not to make diagnostic criteria overly stringent. However, while keeping the index of suspicion for PHACE appropriately high, physicians should consider, as for all patients with unexplained multiple congenital anomalies, testing for other possible diagnoses or sending clinical exome or genome sequencing in addition to chromosomal microarray testing [18]. Because the genetic bases of PHACE (postulated to be due to somatic mosaic mutation) remain unknown, even the establishment of an alternative molecular syndromic diagnosis such as OFCD may not obviate the need to inspect organ systems at risk in PHACE. If the clinical case for PHACE is truly strong, it is possible that some molecular genetic developmental pathways overlap with OFCD or that both diagnoses are appropriate. Thus, clinical judgment after consultation with PHACE experts, and shared decision-making with parents, is needed.

From our phenotypic observations, we hypothesize that BCOR could potentially be a candidate gene for, or in a common pathway disrupted in, PHACE syndrome. A GSEA pathway analysis (described above) indicated that BCOR may be important for embryogenesis and development of the circulatory system and may overlap with genes and/or pathways that have shown to be important in PHACE and/or in infantile hemangioma formation. Currently, the molecular basis of PHACE syndrome is unknown but is suspected to involve somatic mosaic mutation, a phenomenon that has been described in mothers of affected daughters with OFCD [19]. Given that infantile hemangioma is a benign neoplasm that exceedingly rarely transforms to angiosarcoma, along with the recognition that BCOR functions as a tumor suppressor gene implicated in sarcomas as well as some hematologic malignancies (with one reported case of T-cell lymphoma reported in a male with Lenz microphthalmia [4]), systematic investigation of BCOR and PHACE patients may provide important insights into human development and tumorigenesis [20]. BCOR copy number reductions and focal deletions have been reported rarely in retinoblastomas, especially occurring later in childhood [21, 22]. The presence of bilateral multifocal CHRPE, which may warrant continued ophthalmic monitoring, has been reported in one other case of OFCD syndrome [23].

## 5. Conclusion

These two novel cases (one diagnosed with PHACE before OFCD, but both meeting PHACE consensus retrospectively) and two previously published cases, along with potential underreporting, lead us to hypothesize that infantile hemangiomas may be associated with OFCD and other BCOR-related disorders. Due to the phenotypic overlap between OFCD and PHACE, and the results of our gene set enrichment analysis (GSEA), we speculate that BCOR may be a key regulator of development and tumorigenesis, specifically in infantile hemangiomas, and could be implicated in PHACE syndrome. Due to these findings, BCOR should be a target for future genetic studies in infantile hemangiomas and/or PHACE syndrome and/or bilateral CHRPE with congenital cataracts.

## Figures and Tables

**Table 1:** Cross-tabulation of BCOR cases and PHACE criteria.

PHACE criteria (columns to the right) case (row below)	Hemangioma	Arterial anomalies	Structural brain	Cardiovascular	Ocular	Ventral midline	Other features, notably dental anomalies (not described in PHACE criteria)
Previously reported “case 4” in Ragge et al. [4]	(i) Left temporal hemangioma (size not specified)	Absent	Absent	(i) Large ASD	(i) Bilateral posterior embryotoxon	Absent	(i) Large earlobes (ii) Long fingers with 4^th^ and 5^th^ camptodactyly (iii) Short and deep-set toenails (iv) Developmental delay (intellectual disability, speech, and motor delays)

Previously reported “case 8” in Ragge et al. [4]	(i) Large forehead hemangioma (size not specified) (ii) Large neck hemangioma	Absent	Absent	(i) Left ventricular noncompaction (ii) Small persistent ductus arteriosus	(i) Bilateral congenital cataracts (ii) Mild microphthalmia	Absent	(i) Thyroglossal cyst (ii) Agenesis of both superius lateral incisors (iii) Cutaneous syndactyly of second and third toes

Currently reported case 1	(i) Involuted hemangioma of the forehead	Unknown	Unknown	(i) VSD	(i) Bilateral congenital cataracts (ii) CHRPE (iii) Glaucoma	Absent	(i) Dental anomalies (persistent baby teeth, fused baby teeth, delayed eruption of secondary teeth, and long roots of her teeth) (ii) Flat feet (iii) High-arched palate (iv) Facial features that do not resemble other family members (v) Clinodactyly

Currently reported case 2	(i) Right parietal scalp estimated as 5 × 6 cm on physical exam but measuring as 3.9 × 2.3 cm axially by MRI (ii) Smaller lesion within the right frontal scalp measured approximately 1.2 cm in diameter by MRI	(i) Aberrant right subclavian artery	Absent	(i) ASD	(i) Bilateral congenital cataracts (ii) Glaucoma-Microphthalmos left eye	Absent	(i) Long roots of her teeth with one missing tooth and first primary tooth loss at 6-7 years of age (ii) Wolff-Parkinson-White (WPW) syndrome

## Abbreviations

BCOR: BCL-6 corepressor
OFCD: Oculofaciocardiodental syndrome
PHACE: Posterior fossa anomalies, hemangioma, arterial anomalies, cardiac defects, eye anomalies
CHRPE: Congenital hypertrophy of the retinal pigment epithelium
APC: Adenomatous polyposis coli
ASD: Atrial septal defect
VSD: Ventral septal defect
GSEA: Gene set enrichment analysis.

## References

[1] Ng D., Thakker N., Corcoran C. M. (2004). Oculofaciocardiodental and lenz microphthalmia syndromes result from distinct classes of mutations in BCOR. *Nature Genetics*.

[2] Hilton E., Johnston J., Whalen S. (2009). BCOR analysis in patients with OFCD and lenz microphthalmia syndromes, mental retardation with ocular anomalies, and cardiac laterality defects. *European Journal of Human Genetics*.

[3] Gorlin R. J., Marashi A. H., Obwegeser H. L. (1996). Oculo-facio-cardio-dental (OFCD) syndrome. *American Journal of Medical Genetics*.

[4] Ragge N., Isidor B., Bitoun P. (2019). Expanding the phenotype of the X-linked BCOR microphthalmia syndromes. *Human Genetics*.

[5] Frieden I. J., Reese V., Cohen D. (1996). PHACE syndrome: the association of posterior fossa brain malformations, hemangiomas, arterial anomalies, coarctation of the aorta and cardiac defects, and eye abnormalities. *Archives of Dermatology*.

[6] Blair N. P., Trempe C. L. (1980). Hypertrophy of the retinal pigment epithelium associated with Gardner’s syndrome. *American Journal of Ophthalmology*.

[7] Garzon M. C., Epstein L. G., Heyer G. L. (2016). PHACE syndrome: consensus-derived diagnosis and care recommendations. *The Journal of Pediatrics*.

[8] Mootha V. K., Lindgren C. M., Eriksson K. F. (2003). PGC-1alpha-responsive genes involved in oxidative phosphorylation are coordinately downregulated in human diabetes. *Nature Genetics*.

[9] Subramanian A., Tamayo P., Mootha V. K. (2005). Gene set enrichment analysis: a knowledge-based approach for interpreting genome-wide expression profiles. *Proceedings of the National Academy of Sciences of the United States of America*.

[10] Siegel D. H. (2018). PHACE syndrome: infantile hemangiomas associated with multiple congenital anomalies: clues to the cause. *American Journal of Medical Genetics Part C: Seminars in Medical Genetics*.

[11] Nozaki T., Nosaka S., Miyazaki O. (2013). Syndromes associated with vascular tumors and malformations: a pictorial review. *Radiographics*.

[12] Kilcline C., Frieden I. J. (2008). Infantile hemangiomas: how common are they? A systematic review of the medical literature. *Pediatric Dermatology*.

[13] Munden A., Butschek R., Tom W. L. (2014). Prospective study of infantile haemangiomas: incidence, clinical characteristics and association with placental anomalies. *British Journal of Dermatology*.

[14] Coats D. K., Paysse E. A., Levy M. L. (1999). PHACE: a neurocutaneous syndrome with important ophthalmologic implications: case report and literature review. *Ophthalmology*.

[15] ALSomiry A. S., Gregory-Evans C. Y., Gregory-Evans K. (2019). An update on the genetics of ocular coloboma. *Human Genetics*.

[16] Youssef M. J., Siegel D. H., Chiu Y. E., Drolet B. A., Hodgson B. D. (2019). Dental root abnormalities in four children with PHACE syndrome. *Pediatric Dermatology*.

[17] Tortora D., Severino M., Accogli A. (2017). Moyamoya vasculopathy in PHACE syndrome: six new cases and review of the literature. *World Neurosurgery*.

[18] Boycott K. M., Dyment D. A., Innes A. M. (2018). Unsolved recognizable patterns of human malformation: challenges and opportunities. *American Journal of Medical Genetics Part C: Seminars in Medical Genetics*.

[19] Siegel D. H., Shieh J. T., Kwon E. K. (2013). Copy number variation analysis in 98 individuals with PHACE syndrome. *Journal of Investigative Dermatology*.

[20] Kelly M. J., So J., Rogers A. J. (2019). Bcor loss perturbs myeloid differentiation and promotes leukaemogenesis. *Nature Communications*.

[21] Kooi I. E., Mol B. M., Massink M. P. (2016). Somatic genomic alterations in retinoblastoma beyond RB1 are rare and limited to copy number changes. *Scientific Reports*.

[22] McEvoy J., Nagahawatte P., Finkelstein D. (2014). RB1 gene inactivation by chromothripsis in human retinoblastoma. *Oncotarget*.

[23] Zhou Y., Wojcik A., Sanders V. R., Rahmani B., Kurup S. P. (2018). Ocular findings in a patient with oculofaciocardiodental (OFCD) syndrome and a novel BCOR pathogenic variant. *International Ophthalmology*.

